# Performance evaluation of the Alinity m system for quantifying cytomegalovirus DNA in samples of the respiratory, gastrointestinal, and urinary tract

**DOI:** 10.1128/spectrum.04201-23

**Published:** 2024-06-06

**Authors:** Eliseo Albert, Estela Giménez, Juan Alberola, Ignacio Torres, Yolanda López, Ana Marcos, Birgit Reinhardt, David Navarro

**Affiliations:** 1 Hospital Clínico Universitario de Valencia, INCLIVA Health Research Institute, Microbiology Service, Valencia, Spain; 2 Hospital Universitario Doctor Peset, Microbiology Service, Valencia, Spain; 3 Abbott GmbH, Wiesbaden, Germany; 4 Department of Microbiology, School of Medicine, University of Valencia, Valencia, Spain; Johns Hopkins Medicine, Baltimore, Maryland, USA

**Keywords:** nucleic acid amplification test, DNA, cytomegalovirus, immunocompromised, high-throughput diagnostic assay

## Abstract

Quantitation of cytomegalovirus (CMV) DNA load in specimens other than blood such as bronchoalveolar lavages, intestinal biopsies, or urine has become a common practice as an ancillary tool for the diagnosis of CMV pneumonitis, intestinal disease, or congenital infection, respectively. Nevertheless, most commercially available CMV PCR platforms have not been validated for CMV DNA detection in these specimen types. In this study, a laboratory-developed test based on Alinity m CMV (“Alinity LDT”) was evaluated. Reproducibility assessment using spiked bronchial aspirate (BAS) or urine samples showed low standard deviations of 0.08 and 0.27 Log IU/mL, respectively. Evaluating the clinical performance of Alinity LDT in comparison to a laboratory-developed test based on RealTi*m*e CMV (“RealTi*m*e LDT”) showed good concordance across 200 clinical specimens including respiratory specimens, intestinal biopsies, urine, and stool. A high Pearson's correlation coefficient of r = 0.92, a low mean bias of −0.12 Log IU/mL, a good qualitative agreement of 90%, and a Cohen's kappa value of 0.76 (substantial agreement) were observed. In separate analyses of the sample types BAS, tracheal aspirates, bronchoalveolar lavage, biopsies, and urine, the assay results correlated well between the two platforms with r values between 0.88 and 0.99 and a bias <0.5 Log IU/mL. Overall, the fully automated, continuous, random access Alinity LDT yielded good reproducibility, high concordance, and good correlation to RealTi*m*e LDT in respiratory, gastrointestinal, and urine samples and may enhance patient management with rapid result reporting.

**IMPORTANCE:**

In transplant recipients, a major cause for morbidity and mortality is end-organ disease by primary or secondary CMV infection of the respiratory or gastrointestinal tract. In addition, sensorineural hearing loss and neurodevelopmental abnormalities are frequent sequelae of congenital CMV infections in newborns. Standard of care for highly sensitive detection and quantitation of the CMV DNA load in plasma and whole blood specimens is real-time PCR testing. Beyond that, there is a need for quantitative determination of CMV DNA levels in respiratory, gastrointestinal, and urinary tract specimens using a highly automated, random access CMV PCR assay with a short turnaround time to enable early diagnosis and treatment. In the present study, clinical performance of the fully automated Alinity m analyzer in comparison to the current RealTi*m*e LDT assay was evaluated in eight different off-label sample types.

## INTRODUCTION

Cytomegalovirus (CMV) infection continues to be a frequent cause of morbidity and mortality in transplant recipients (1–3). Real-time PCR has emerged as a highly sensitive approach for quantifying CMV DNA in plasma or whole blood specimens, which is pivotal in the management of CMV infection in these patients (2, 4).

In immunocompromised patients, potentially life-threatening end-organ disease as pneumonitis can be caused by CMV (3, 5). Clinical utility of CMV quantitation in selective deep respiratory samples as bronchoalveolar lavage (BAL) is based on a high negative predictive value (NPV) of almost 100% while the positive predictive value for CMV pneumonitis increases with higher CMV DNA load in BAL as well as with a higher patient risk for CMV disease. The value of CMV DNA quantitation in BAL may allow discrimination between asymptomatic CMV shedding in the absence of a proven disease and CMV pneumonitis (6, 7).

CMV infection in the gastrointestinal tract can lead to the development and relapse of inflammatory bowel disease (IBD) in immunocompromised patients (8, 9). Moreover, gastrointestinal CMV disease is present in 70%–80% of all CMV disease cases among hematopoietic stem cell transplant (HSCT) recipients and is often associated with graft-versus-host disease (6). In order to diagnose gastrointestinal CMV disease, assessment of gastrointestinal biopsies is preferred over plasma or whole blood as CMV replication may be compartmentalized to occur in the gastrointestinal mucosa (6). In comparison to immunohistochemistry (IHC) as the gold standard, CMV DNA determination in biopsies showed high sensitivity and a NPV of 100% (9). Thus, the European Crohn's and Colitis Organization guidelines recommend CMV PCR testing as an option besides IHC to confirm active CMV infection (10). Sensitive CMV DNA quantification allows monitoring of treatment response in the patient, albeit consensus CMV DNA cut-off values to determine CMV disease, initiate CMV-specific treatment, or predict refractoriness to certain drug therapies still remain to be established (8).

Congenital CMV infection in newborns is a leading cause of sensorineural hearing loss and neurodevelopmental abnormalities. For diagnosis of congenital CMV infection, it is recommended to test urine or saliva specimens of neonates with nucleic acid tests during their first 3 weeks of life, since early treatment of CMV-infected newborns is associated with significant improvement in outcomes (11).

Rapid or conventional cell-culture-based assays performed on biopsies or other specimen types remain the gold standard for diagnosis of congenital CMV infections or CMV end-organ disease involving the lungs or the gastrointestinal tract (12). However, traditional culture needs long time to results, is labor-intensive, and requires skilled personnel (13, 14). The limitations of culture technique prompted the development of nucleic acid tests as laboratory-developed tests (LDTs). Many commercially available automated quantitative PCR tests, such as the Abbott RealTi*m*e CMV and the Alinity m CMV assays (both Abbott, Des Plaines, IL, USA), are validated only for whole blood and plasma samples (15, 16). Because of the clinical significance of detecting and monitoring CMV DNA in non-blood compartments of the patients, our institutions had validated the Abbott RealTi*m*e CMV assay on the semi-automated *m*2000 platform as a laboratory-developed test (“RealTi*m*e LDT”) for these sample types and are currently using it for routine testing. However, the *m*2000 system only allows batchwise testing that requires sorting and storage of samples. In contrast, the fully automated, high-throughput, continuous Alinity m platform provides a streamlined, random access workflow independent of the assay, with a processing turnaround time of less than 2 hours, thus enabling earlier diagnosis and treatment (17).

The objective of the present study was to evaluate the clinical performance of this fully automated Alinity m analyzer in measuring CMV DNA levels in respiratory, gastrointestinal, and urine samples (“Alinity LDT”) in comparison to the current RealTi*m*e LDT and to assess the reproducibility of Alinity LDT in two selected sample types.

## MATERIALS AND METHODS

### Study sites

The study was performed at the Microbiology Service of the Hospital Clínico Universitario de Valencia, INCLIVA Health Research Institute, and at the Hospital Dr. Peset, both located in Valencia, Spain. The investigation was conducted according to the principles of the Declaration of Helsinki, and an approval by an ethics committee was obtained by the Institutional Review Board of the Hospital Clínico Universitario de Valencia.

### CMV testing

RealTi*m*e LDT was tested on the *m*2000 system (Abbott Molecular, Des Plaines, IL, USA) using the plasma protocol of the RealTi*m*e CMV assay (15). The process consists of sample extraction on the *m*2000sp and subsequent real-time PCR amplification and detection on the *m*2000rt. The *m*2000 system uses a semi-automated batch workflow and can process up to 96 samples in approximately 8 hours.

Alinity LDT was tested on the Alinity m system (Abbott Molecular Inc., Des Plaines, IL, USA), applying the plasma protocol of the Alinity m CMV assay (16). The Alinity m analyzer can store 20 reagent packs on board, process 300 samples in a single 8-hour shift, and allows prioritization of urgent samples using the STAT functionality.

Both assays target the same genomic sequence within the highly conserved UL34 and UL80.5 genes.

### Reproducibility assessment

The reproducibility of Alinity LDT was assessed by testing a negative bronchial aspirate (BAS) patient sample spiked with a CMV DNA positive BAS sample and a negative urine patient sample spiked with a CMV DNA positive urine sample. CMV DNA in the BAS and urine samples was value assigned on the RealTi*m*e LDT to 2.8 Log IU/mL and 3.4 Log IU/mL, respectively. Samples were processed using Alinity LDT in up to five replicates, run on five consecutive days, for a total of up to 25 replicates for each specimen type.

### Clinical specimens and evaluation of the clinical performance

Two hundred de-identified, non-consecutive surplus patient specimens from routine diagnostic testing at the Hospital Clínico Universitario de Valencia (*n* = 165) or at the Hospital Dr. Peset (*n* = 35) were retrospectively selected with no inclusion or exclusion criteria applied. Demographics and clinical characteristics of the patients are summarized in Table 1. The collected specimen types included BAS (*n* = 47), tracheal aspirate (TA, *n* = 25), bronchoalveolar lavage (BAL, *n* = 41), bronchial brushing (*n* = 4), pleural fluid (*n* = 2), intestinal biopsies (*n* = 42), urine (*n* = 34), and stool samples (*n* = 5).

**TABLE 1:** Demographics and clinical characteristics of the study population^*a*^

Number of patients (*n*; %)	200 (100%)
Sex	
Male (*n*; %)	123 (61.5%)
Female (*n*; %)	77 (38.5%)
Age	
Median (IQR)	58 (41–69)

Baseline disease (*n*; %)	
Hematological disease	
Allogenic-HSCT	16 (8.0%)
Autologous-HSCT	1 (0.5%)
Lymphoma	4 (2.0%)
Leukemia	5 (2.5%)
Other	4 (2.0%)
Other neoplastic diseases	
Carcinomas	13 (6.5%)
Other non-neoplastic causes	
Respiratory pathology (pneumonia/bronchiectasis)	65 (32.5%)
Congenital CMV	30 (15.0%)
Inflammatory bowel disease	28 (14.0%)
Other	34 (17.0%)

^*a*^ IQR: Interquartile range; HSCT: hematopoietic stem cell transplantation.

As pretreatment prior to routine testing, BAS and TA samples with a high viscosity or a high content of mucus were mixed with the lowest possible amount of dithiothreitol (Sputasol, Oxoid Ltd., Basingstoke, United Kingdom), typically not exceeding the sample volume, to obtain a liquid and homogeneous consistency (18, 19). Biopsies were manually homogenized in a sterile mortar with 1–2 mL of sterile saline and transferred into a sterile container using a sterile Pasteur pipette (20). Stool samples were centrifuged, and the supernatant was used for testing. If necessary, a small volume of water was added first for a dilution of maximum 1:10, and the stool sample was vortexed prior to centrifugation. All pretreatment methods had been laboratory-internally validated to ensure reliable results. Urine, BAL, pleural fluids, and bronchial brushings (immersed in physiological saline solution) were analyzed without any pretreatment. All specimens had been stored frozen at −80°C for 2–24 months after clinical routine analysis.

For the study, samples were thawed, vortexed, and aliquoted for analysis on both platforms at the respective study site. Overall, 200 clinical specimens were tested in parallel with Alinity LDT and RealTi*m*e LDT for the presence of CMV DNA. The concordance of the assay results was determined.

### Statistical analysis

Mean, standard deviation, and coefficient of variation (CV) were determined for within-day, between-day, and total reproducibility of Alinity LDT in bronchial aspirate and urine using the software JMP 15.2.1 with the module FitModel. For the comparison of Alinity LDT and RealTi*m*e LDT, the software R version 3.5.3 was used to calculate Deming regression with Pearson's correlation coefficient r as well as mean bias and mean standard deviation (Bland-Altman analysis).

## RESULTS

### Reproducibility assessment

Reproducibility analysis using spiked BAS and urine samples resulted in an overall mean concentration of 2.83 and 3.29 Log IU/mL, a low overall SD of 0.08 and 0.27 Log IU/mL, and overall %CVs of 2.9% and 8.2%, respectively (Table 2). The agreement with the expected values was high. The differences in quantitation (mean-target concentration) were below 0.5 Log IU/mL with 0.03 Log IU/mL in BAS and −0.11 Log IU/mL in urine.

**TABLE 2:** Reproducibility of CMV DNA quantified by Alinity LDT using bronchial aspirate (BAS) and urine samples^*a*^

Specimen type	*N*	Target Conc. (Log IU/mL)	Mean Conc. (Log IU/mL)	Difference Mean-Target (Log IU/mL)	Within-Day Component		Between-Day Component		Total	
					SD (Log IU/mL)	CV (%)	SD (Log IU/mL)	CV (%)	SD (Log IU/mL)	CV (%)
BAS	22	2.80	2.83	0.03	0.08	2.9	0.00	0.0	0.08	2.9
Urine	25	3.40	3.29	−0.11	0.21	6.4	0.17	5.1	0.27	8.2

^*a*^ SD: Standard deviation; CV: Coefficient of variation.

### Clinical assessment

A total of 200 clinical specimens were tested by Alinity LDT and RealTi*m*e LDT including 119 respiratory samples (47 BASs, 25 TAs, 41 BALs, 4 bronchial brushings, and 2 pleural fluids), 42 biopsies, 34 urine samples, and 5 stool specimens. Deming regression analysis indicated high concordance between Alinity LDT and RealTi*m*e LDT across all 200 specimens (Pearson's correlation coefficient r = 0.92; Fig. S1A) with a mean bias of −0.12 Log IU/mL (Alinity - RealTi*m*e) (Fig. S1B). The overall qualitative agreement, calculated as concordant negative and positive [below lower limit of quantitation (<LLOQ) or quantitated] results, was 90% (Table S1) with a Cohen's kappa value of 0.76 representing substantial agreement (21). Twenty-one discordant results were observed of which 20 had weak CMV DNA loads ≤ 1.66 Log IU/mL with one assay and “not detected” results with the other test. Only one biopsy specimen had a “not detected” result with RealTi*m*e LDT but a CMV DNA load of 5.49 Log IU/mL with Alinity LDT. Retesting with Alinity LDT confirmed the previous result (5.14 Log IU/mL). There was not sufficient sample volume available for further analysis.

Of the 100 specimens quantified by both methods, in 80 samples, the difference in quantitation was <0.5 Log IU/mL; in 13 samples, the difference was between 0.5 and 1.0 Log IU/mL; and in 7 samples, it was above 1.0 Log IU/mL. Interestingly, these seven samples included five biopsy specimens.

**Fig 1:**
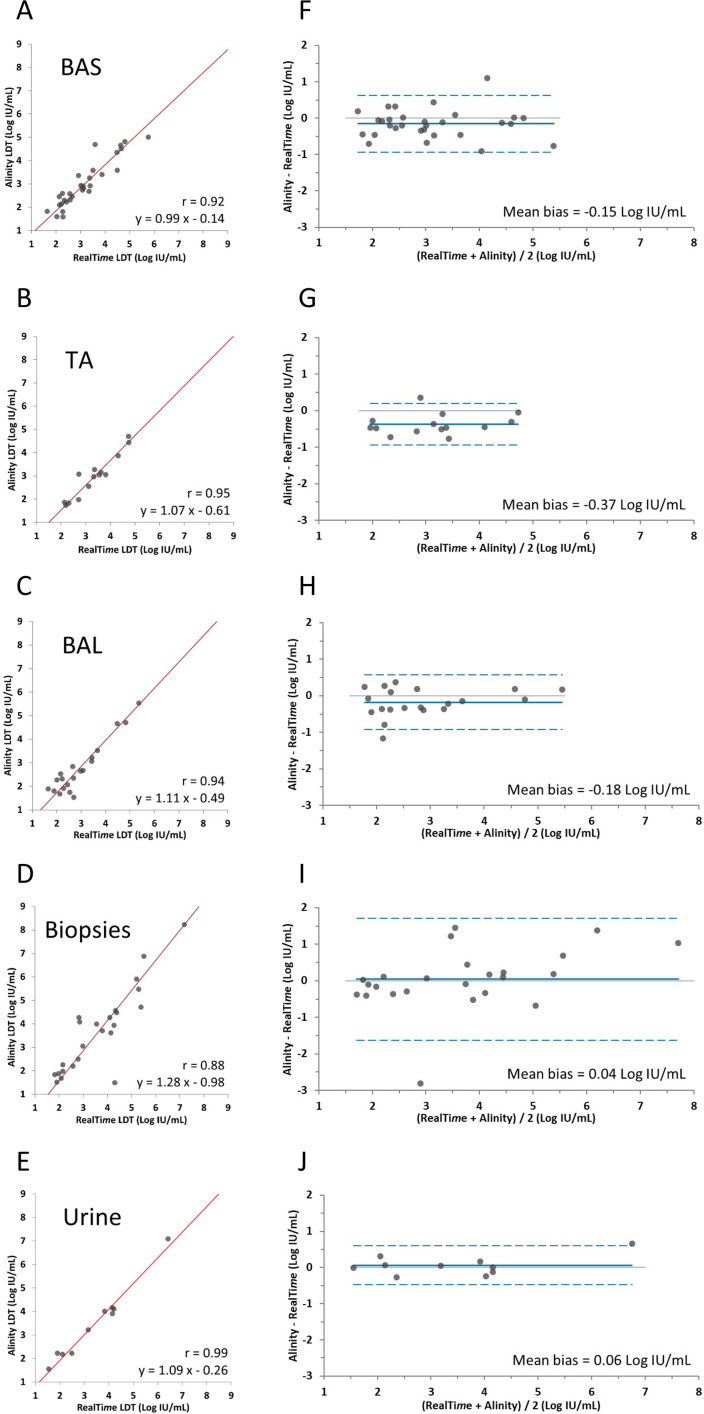
Correlation and Deming regression comparing Alinity LDT and RealTi*m*e LDT assay results in five different sample types with at least 10 samples quantified by both assays: (**A**) BAS (*n* = 30), (**B**) TA (*n* = 14), (**C**) BAL (*n* = 20), (**D**) biopsies (*n* = 24), and (**E**) urine (*n* = 10). Bland-Altman analysis: (**F**) BAS, (**G**) TA, (**H**) BAL, (**I**) biopsy, and (**J**) urine.

Separate Deming analyses were conducted for five specimen types that contained at least 10 samples with quantified results by both assays: 30 BAS, 14 TA, 20 BAL, 24 biopsy, and 10 urine samples. Evaluation revealed high concordance with Pearson's correlation coefficients r close to 1 for urine (r = 0.99) and the respiratory specimens TA (r = 0.95), BAL (r = 0.94), and BAS (r = 0.92), while the r value was slightly lower for biopsies (r = 0.88; Fig. 1A through E). Bland-Altman analysis demonstrated a low mean bias between −0.37 and +0.06 Log IU/mL (Alinity - RealTi*m*e) for all five specimen types (Fig. 1F through J). The overall qualitative agreement and Cohen's kappa values were 93.6% and 0.69 for BAS, 92.0% and 0.80 for TA, 87.8% and 0.72 for BAL, 86.0% and 0.65 for biopsies, and 91.2% and 0.82 for urine, respectively (Table 3A through E). Cohen's kappa values represented almost perfect agreement for urine and substantial agreement for the other four sample types. For the remaining samples (four bronchial brushing specimens, two pleural fluid samples, and five stool samples), either “not detected” results by one or both methods or weak CMV DNA loads of less than or equal to 3.21 Log IU/mL were obtained. Differences in quantitation were low with a maximum of 0.37 Log IU/mL (Table 4).

**TABLE 3:** Concordance of CMV DNA results obtained with Alinity LDT and RealTi*m*e LDT across different specimen types with at least 25 samples tested: BAS, TA, BAL, biopsies, and urine^*a*^

BAS		RealTi*m*e LDT			Total
		Quantified	<LLOQ	Not Detected	
Alinity LDT	Quantified	30	1	2^*b*^	33
	<LLOQ	5	4	1	10
	Not detected	0	0	4	4
Total		35	5	7	47

TA		RealTi*m*e LDT			Total
		Quantified	<LLOQ	Not Detected	
Alinity LDT	Quantified	14	0	0	14
	<LLOQ	3	0	0	3
	Not detected	0	2	6	8
Total		17	2	6	25

BAL		RealTi*m*e LDT			Total
		Quantified	<LLOQ	Not Detected	
Alinity LDT	Quantified	20	1	0	21
	<LLOQ	2	2	2	6
	Not detected	0	3	11	14
Total		22	6	13	41

Biopsies		RealTi*m*e LDT			Total
		Quantified	<LLOQ	Not Detected	
Alinity LDT	Quantified	24	0	1^*d*^	25
	<LLOQ	1	2	2	5
	Not detected	1^*c*^	2	9	12
Total		26	4	12	42

Urine		RealTi*m*e LDT			Total
		Quantified	<LLOQ	Not Detected	
Alinity LDT	Quantified	10	1	0	11
	<LLOQ	0	1	3	4
	Not detected	0	0	19	19
Total		10	2	22	34

^*a*^ The lower limit of quantification (LLOQ) of the used Alinity m CMV and RealTi*m*e CMV plasma protocols is 1.48 Log IU/mL and 1.70 Log IU/mL, respectively.

^*b*^ 2 specimens with CMV DNA not detected by RealTi*m*e LDT were quantitated at 1.49 and 1.66 Log IU/mL with Alinity LDT.

^*c*^ 1 specimen with CMV DNA not detected by Alinity LDT was quantitated at 1.55 Log IU/mL with RealTi*m*e LDT.

^*d*^ 1 specimen with CMV DNA not detected by RealTi*m*e LDT was quantitated at 5.49 Log IU/mL with Alinity LDT.

**TABLE 4:** Method comparison for specimen types with less than 25 samples

Specimen type	*n*	RealTi*m*e LDT (CMV DNA Log IU/mL)	Alinity LDT (CMV DNA Log IU/mL)	Difference (Alinity-RealTi*m*e) (CMV DNA Log IU/mL)
Bronchial brushing	2	Not detected	Not detected	n/a
	1	1.54	Not detected	n/a
	1	2.25	1.89	−0.37
Pleural fluid	1	1.98	<1.48	n/a
	1	3.21	3.08	−0.13
Stool	4	Not detected	Not detected	n/a
	1	<1.49	Not detected	n/a

## DISCUSSION

This is one of the first studies to provide evidence of the utility of Alinity LDT to quantify CMV DNA in respiratory, gastrointestinal, and urine specimen types and to assess concordance with the corresponding laboratory-validated RealTi*m*e LDT results.

Reproducibility was high when testing up to 25 replicates of spiked BAS and urine samples, respectively, with Alinity LDT. The observed total SD of ≤0.27 Log IU/mL and the low difference in quantitation versus the target concentration (≤0.11 Log IU/mL) were comparable to those recently reported for Alinity m CMV in plasma (SD ≤0.28 Log IU/mL; difference in quantitation to the expected value ≤0.12 Log IU/mL) (22).

Urine results correlated well between Alinity LDT and RealTi*m*e LDT with a high Pearson's correlation coefficient r = 0.99, a low mean bias of 0.06 Log IU/mL, and a kappa value of 0.82 representing almost perfect agreement. In a previous study, RealTi*m*e CMV had been laboratory-validated for urine samples. The limit of detection was determined at 170 IU/mL. High precision and reproducibility were observed (SD values ≤0.18 Log IU/mL). Additionally, the clinical comparison of RealTi*m*e CMV to viral culture in urine samples of newborns yielded high concordance (kappa value, 0.96), 100% sensitivity, and 98.2% specificity (23). Based on these excellent validation results for the comparator RealTi*m*e LDT, the Alinity LDT results look very promising.

In addition, 119 deep respiratory samples including BAS, TA, BAL, bronchial brushing, and pleural fluids were tested by Alinity LDT in parallel to RealTi*m*e LDT. Evaluation of BAS, TA, and BAL revealed high correlation with a Pearson's correlation coefficient r of 0.92–0.95 and an overall qualitative agreement of 87.8%–93.6%. Discrepant results were only observed at low CMV DNA levels. A correlation with the clinical condition of the patients or establishment of a clinical cut-off to identify patients with CMV pneumonia was beyond the scope of this study. However, another small clinical study had correlated RealTi*m*e CMV results in BAL to patients' diagnosis and outcome. Based on 11 patients who developed CMV pneumonitis, the authors suggested a CMV DNA load of 4.39 Log IU/mL as optimal cut-off value to discriminate between patients with CMV pneumonia and asymptomatic pulmonary viral shedding (24). In a larger study evaluating BAL specimens of 61 patients with RealTi*m*e CMV, a median of 2.54 Log IU/mL was observed for patients without CMV pneumonia, while in a second study center with a different assay, a median of 3.29 Log IU/mL was determined (5). Previously, a clinical threshold of >500 IU/mL using a laboratory-developed CMV PCR assay was suggested to differentiate patients with CMV pneumonia from asymptomatic pulmonary shedding in HSCT recipients (7). Another small study compared RealTi*m*e CMV as reference assay to Roche CAP/CTM CMV evaluating 17 BAL samples. Fourteen samples were quantified by both assays resulting in a mean bias of 0.93 Log IU/mL (CAP/CTM - RealTi*m*e) (25). Overall, a number of influencing factors including pulmonary hemorrhage, preceding antiviral therapy, amount of cells in the BAL sample, CMV prevalence, or quantitation differences between PCR assays as shown above make interpretation of CMV DNA loads in BAL challenging and complicate the definition of a universal threshold (5). Nevertheless, as the mean bias between Alinity LDT and RealTi*m*e LDT is only −0.18 Log IU/mL, similar clinical cut-off ranges might be expected for Alinity LDT in future studies.

In the present study, the Pearson's correlation coefficient in biopsies (r = 0.88) was similar to those in urine and respiratory specimens albeit slightly lower. Furthermore, five of seven samples with a difference in quantitation >1 Log IU/mL were biopsies as well as a discrepant sample with 5.49 Log IU/mL by Alinity LDT but a “not detected” result by RealTi*m*e LDT. Inhomogeneous distribution of CMV-infected cells within the biopsy sample together with the manual pretreatment procedure for biopsy specimens may have caused non-uniform distribution of CMV DNA load in the specimens that could have led to the observed variations. In the case of the discrepant sample, any impact by potential mutations can be excluded as both assays use the same target regions. Also a failed pretreatment does not appear to be probable as aliquoting for the two platforms was performed subsequently. Furthermore, during previous routine testing with RealTi*m*e LDT, a CMV DNA load of 6.28 Log IU/mL had been observed which confirmed the later Alinity LDT result. Thus, a mix-up of samples or a storage issue after aliquoting cannot be excluded. Unfortunately, there was no sample volume left to perform additional analyses. The other discrepant biopsy samples showed only weak CMV DNA loads below or equal to 1.55 Log IU/mL.

Studies had shown that histopathologically positive biopsies were associated with a significantly higher median CMV DNA load than biopsies with a negative histopathological result. Therefore, it has been recommended to establish a cut-off value for CMV DNA load in biopsies to distinguish CMV intestinal disease from the detection of latent CMV DNA (26).

Five stool samples included in the present study showed negative or weak positive CMV DNA results. Similarly, other studies using RealTi*m*e CMV or another assay observed rare and low CMV DNA loads in stool specimens suggesting that, even in cases of CMV intestinal disease, only limited amounts of CMV DNA are shed into feces (27, 28).

One limitation of our study was the use of a reference assay (RealTi*m*e LDT) for which the evaluated sample types were not validated by the manufacturer. However, due to a lack of approved commercial assays, it is very common to laboratory-internally validate a CMV assay that is CE-marked for plasma and/or whole blood. Another limitation was the small number of samples for some specimen types that limited statistical evaluation. Moreover, since de-identifed samples were evaluated, a correlation to the patient history was not possible. However, the focus of this study was on the correlation of Alinity LDT with the established laboratory-validated RealTi*m*e LDT assay, which was good. Another limitation of this study was that, in biopsies, cell distribution was not controlled by normalizing the cell number, e.g. by measuring the human CRP gene with a second real-time PCR (26). This may have reduced the observed variability to a similar level as in the liquid sample types. Additionally, general data regarding reproducibility of CMV DNA quantitation in biopsies is scarce. Further limitations of our study included the use of only two sample types, urine and BAS, for the reproducibility assessment. Although urine and lower respiratory tract samples represented the majority of specimens besides plasma samples, reproducibility may vary across different sample types. Also pretreatment could have introduced variability into results and potentially impacted sensitivity. Quantitation was not affected by the pretreatment as results were calculated for the undiluted samples. Finally, additional analyses of discrepant results were not possible due to the lack of residual sample volume; though in most cases, discrepancies only occurred at low CMV DNA loads.

In conclusion, Alinity LDT was able to quantify CMV DNA in respiratory, gastrointestinal, and urine sample types yielding similar results as RealTi*m*e LDT, thereby expanding its utility in differential diagnosis of localized vs systemic infections. Rapid reporting of CMV assay results with fully automated systems such as the Alinity m can help improve decision making and patient care.

## SUPPLEMENTAL MATERIAL

Figure S1 spectrum.04201-23-s0001.docx Comparison between Alinity LDT and RealTi*m*e LDT results.

Table S1 spectrum.04201-23-s0002.docx Qualitative concordance of Alinity LDT and RealTi*m*e LDT results.

ASM does not own the copyrights to Supplemental Material that may be linked to, or accessed through, an article. The authors have granted ASM a non-exclusive, world-wide license to publish the Supplemental Material files. Please contact the corresponding author directly for reuse.
