# Supplementary material for: Performance evaluation of the Alinity m system for quantifying cytomegalovirus DNA in samples of the respiratory, gastrointestinal, and urinary tract
Source: Microbiol Spectr. 2024 Jun 6;12(7):e04201-23. doi: 10.1128/spectrum.04201-23 (PMC11218520; doi:10.1128/spectrum.04201-23)
Supplement: Figure S1 — Comparison between Alinity LDT and RealTime LDT results. [file spectrum.04201-23-s0001.docx]

**Supplemental Figure S1**

**A**

**
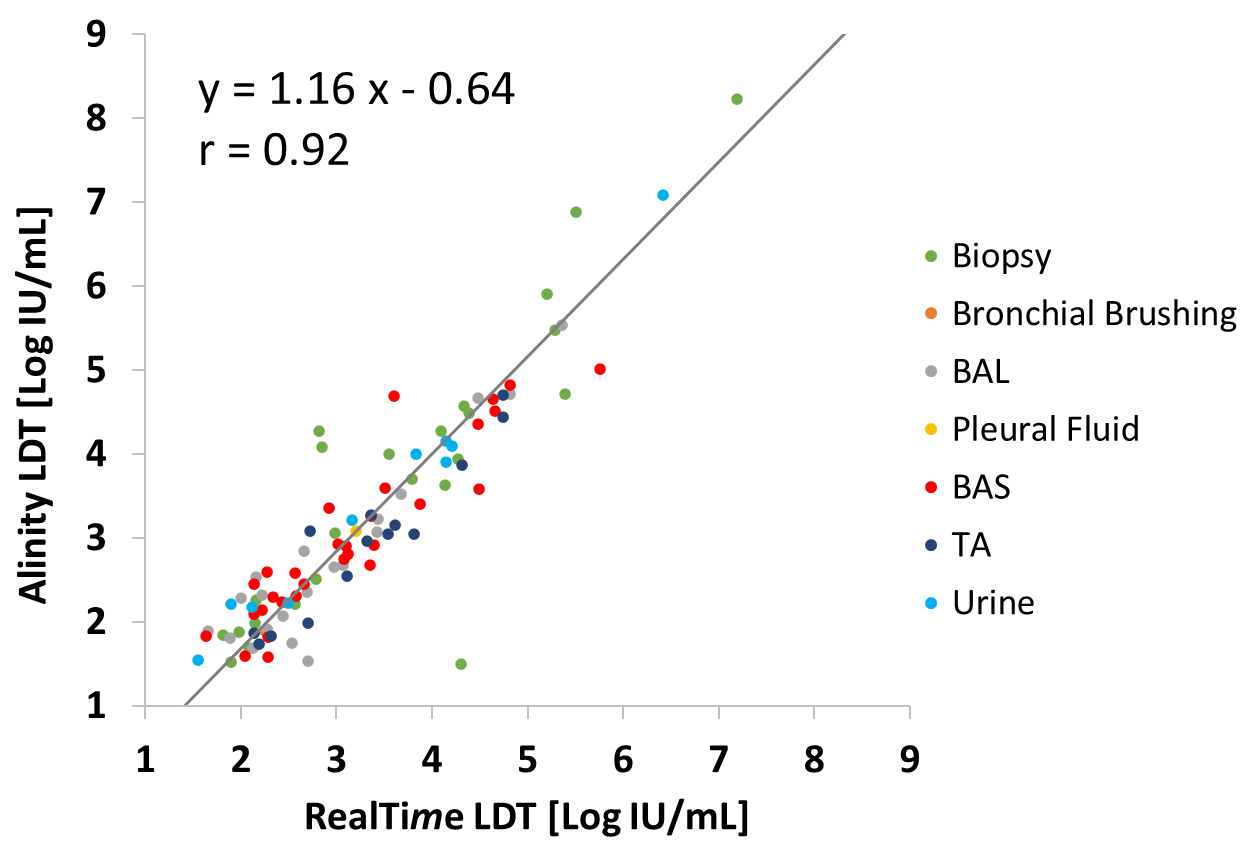
**

**B**

**
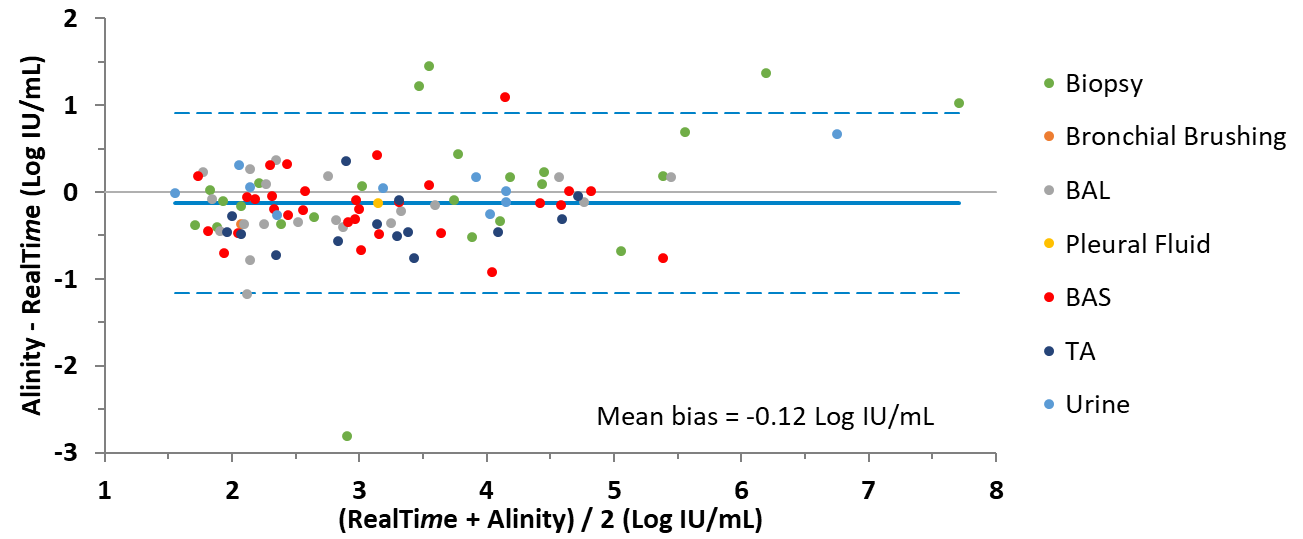
**

**Figure S1:** Comparison between Alinity LDT and **RealTi*m*e** LDT results across seven different sample types with quantified CMV DNA results by both assays (n=100): (A) Deming regression with a high Pearson’s correlation coefficient (r = 0.92) and (B) Bland-Altman graph with a low mean bias (‑0.12 Log IU/mL).
