# Supplementary material for: Performance evaluation of the Alinity m system for quantifying cytomegalovirus DNA in samples of the respiratory, gastrointestinal, and urinary tract
Source: Microbiol Spectr. 2024 Jun 6;12(7):e04201-23. doi: 10.1128/spectrum.04201-23 (PMC11218520; doi:10.1128/spectrum.04201-23)
Supplement: Table S1 — Qualitative concordance of Alinity LDT and RealTime LDT results. [file spectrum.04201-23-s0002.docx]

**Performance evaluation of the Alinity m system for quantifying cytomegalovirus DNA in samples of the respiratory, gastrointestinal, and urinary tract**

Eliseo Albert^a#^, Estela Giménez^a^, Juan Alberola^b^, Ignacio Torres^a^, Yolanda López^a^, Ana Marcos^a^, Birgit Reinhardt^c^, David Navarro^a,d^

**Supplemental Material**

**Supplemental Tables**

**Table S1:** Qualitative concordance of Alinity LDT and RealTi*m*e LDT results across all 200 tested specimens of the different specimen types. Overall qualitative agreement was 90%, and the Cohen’s kappa value was 0.76 representing substantial agreement.

| **All specimens** | | **RealTi*m*e LDT** | | **Total** |
| --- | --- | --- | --- | --- |
|  |  | **Positive** | **Negative** |  |
| **Alinity LDT** | **Positive** | 124 | 11^†^ | **135** |
|  | **Negative** | 10* | 55 | **65** |
| **Total** | | **134** | **66** | **200** |

*10 specimens with CMV DNA not detected by Alinity LDT were quantitated at 1.54 and 1.55 Log IU/mL or detected positive < LLOQ with RealTi*m*e LDT

^†^ 11 specimens with CMV DNA not detected by RealTi*m*e LDT were quantitated at 1.49, 1.66, and 5.49 Log IU/mL or detected positive < LLOQ with Alinity LDT

The lower limit of quantification (LLOQ) of the used Alinity m CMV and RealTi*m*e CMV plasma protocols is 1.48 Log IU/mL and 1.70 Log IU/mL, respectively.
